# Structural Modification of Phenolic Acids: Modern Approaches to Synthesis and Study of Structure and Activity

**DOI:** 10.3390/ijms27072977

**Published:** 2026-03-25

**Authors:** Almagul K. Umbetova, Yuliya A. Litvinenko, Meruyert D. Dauletova, Li Yi, Nazym S. Yelibayeva, Gauhar Sh. Burasheva, Larissa R. Sassykova, Aisulu Zh. Kabdraisova, Zhanibek S. Assylkhanov, Subramanian Sendilvelan, Sergey N. Kalugin, Kannayiram Gomathi, Ruimao Hua

**Affiliations:** 1 Department of Chemistry and Technology of Organic Substances, Natural Compounds and Polymers, Faculty of Chemistry and Chemical Technology, Al-Farabi Kazakh National University, 71 Al-Farabi Ave., Almaty 050040, Kazakhstan; almagulu15@gmail.com (A.K.U.); ly2529459693@gmail.com (L.Y.); nazym_yelibaeva@mail.ru (N.S.Y.); gauharbur@mail.ru (G.S.B.); zhanik1903@list.ru (Z.S.A.); kalugin_sn_org@mail.ru (S.N.K.); 2 Faculty of Chemistry and Chemical Technology, Al-Farabi Kazakh National University, 71 Al-Farabi Ave., Almaty 050040, Kazakhstan; larissa.rav@mail.ru; 3 Scientific Research Institute for New Chemical Technologies and Materials, Al-Farabi Kazakh National University, 96 Tole Bi Str., Almaty 050012, Kazakhstan; zhaksais@gmail.com; 4 Department of Mechanical Engineering, Dr.M.G.R. Educational and Research Institute, Chennai 600095, India; sendilvelan.mech@drmgrdu.ac.in; 5 Department of Biotechnology, Dr.M.G.R. Educational and Research Institute, Chennai 600095, India; gomathi.ibt@drmgrdu.ac.in; 6 Department of Chemistry, Tsinghua University, 30 Shuangqing Rd, Haidian District, Beijing 100084, China; ruimao@mail.tsinghua.edu.cn

**Keywords:** phenolic acids, benzoic series, cinnamic series, structural modification, terrestrial ecosystem, biological activity, drug compounds, public health, green chemistry, pharmacological potential

## Abstract

The paper discusses a comprehensive analysis of contemporary approaches to the structural modification of phenolic acids from the benzoic (C_6_–C_1_) and cinnamic (C_6_–C_3_) series, which have a wide range of biological activity. The main directions of chemical transformation of phenolic compounds that aim to improve their pharmaco-logical potential, stability, and bioavailability are discussed. The given study also makes an accent on the relationship between the structural features and biological effects of various phenolic acid derivatives, including antioxidant, anti-inflammatory, antimicrobial, and antitumor activity. The purpose of this investigation is to systematize current data on strategies for the structural modification of phenolic acids, to identify key areas of their chemical transformation, and to determine the most promising methods for creating biologically active compounds with improved pharmacological properties. The study was aimed at systematizing the accumulated knowledge and identifying promising areas for further research in the field of structural design of phenolic acid derivatives. Based on the analysis of experimental data and literature sources, key trends in the development of new medicinal compounds using the example of natural phenolic acids were characterized, and prospects for their use in medicine and pharmaceutical chemistry were shown.

## 1. Introduction

Phenolic acids are a common type of natural aromatic compounds. The fundamental characteristic of these substances is the presence of one or more hydroxyl groups on the benzene ring and due to this structure phenolic acids have their significant antioxidant activity and various biological effects [1,2].

In terms of structure, phenolic acids are mainly divided into two major categories: benzoic acid type (C_6_–C_1_) and cinnamic acid type (C_6_–C_3_) derivatives. They often occur in plants and play an important role in plant metabolism. At the same time, they are also important precursors for the synthesis of various plant secondary metabolites [3].

In recent decades, researchers have paid increasing attention to phenolic acids. It is believed that phenolic acids are a type of drug structural unit with potential applications. Their rich structural types, high chemical reactivity, and inherent biological activity make them suitable for developing compounds with antioxidant, anti-inflammatory, antibacterial, and anti-tumor properties [4]. Moreover, phenolic acids occur in large quantities in nature and have low toxicity which imparts them with great value in drug chemistry and biomedical research.

Although there have been many studies, the application of phenolic acids still has some problems. Many phenolic acids have low bioavailability in the body. Under physiological conditions, these compounds are not very stable. They also have poor ability to cross the cell membrane. To address these issues, researchers usually use chemical modification methods to improve the pharmacokinetic and pharmacodynamic properties of phenolic acids. However, current research on the relationship between structural changes and biological activity has not been systematically summarized yet. This limits the further development of phenolic acids.

With the development of synthetic chemistry, catalytic technology and biotechnology, new opportunities for the study of phenolic acid structure modification started emerging. Researchers can modify phenolic acids through esterification, acylation, and amidation. They can also introduce new aromatic rings or heterocyclic structures, or construct hybrid molecules. These methods can effectively upgrade the structural types of phenolic acid derivatives [5]. However, the current research results are scattered across different literature and there is a lack of systematic summaries. Therefore, there is an urgent necessity in conducting a comprehensive and updated analysis of phenolic acids and their derivatives have practical significance and research value.

The objective of the study is to classify available data on the chemical transformation of benzoic and cinnamic acid derivatives, to summarize recent advances in their structural modification, and to identify key patterns determining their biological activity and potential as drug candidates. The following issues are discussed: classification and natural distribution of phenolic acids; the main types of structural modifications and strategies of molecular design; the impact of these modifications on physico-chemical and biological properties; and current trends and promising areas of further research aimed at creating new pharmacologically active molecules based on phenolic acids.

## 2. Methodology

The chemical structures of phenolic acid derivatives presented in this work were constructed using ChemDraw Professional 20.0 and Chem3D 20.0 software (PerkinElmer, Shelton, CT, USA). Two-dimensional structures of the compounds were drawn on the basis of published literature data related to the synthesis, structural modification, and biological activity of benzoic acid and cinnamic acid derivatives. The selection of structures was carried out according to reported natural sources, synthetic routes, and pharmacological studies described in the literature.

Three-dimensional (3D) models of selected molecules were generated by molecular modeling using Chem3D software, followed by geometry optimization with the standard molecular mechanics tools included in the program. The obtained molecular models are used to display the spatial distribution of functional groups, the molecular conformation, and the spatial steric hindrance effects that may influence intermolecular interactions and biological activity. Through the visualization of the three-dimensional structure, a better understanding of the relationship between molecular structure and biological activity can be achieved. Therefore, this method has been widely applied in modern drug chemistry and computer-aided drug design research [6].

The construction method of the molecular structure in this article is based on the published research works. These studies introduced molecular modeling techniques and the applications of ChemDraw and Chem3D software in chemical and drug research. All the chemical structures shown in the tables and figures in this article were redrawn by the author to ensure the consistency of the presentation format throughout the text. The numbering of the compounds in this article is organized according to the unified system of this article, so it does not exactly correspond to the numbering in the original literature.

The structural diagrams and molecular models presented in this article are intended to illustrate the structural diversity of phenolic acid derivatives, and to provide a foundation for discussing their chemical reactivity, biological activity, and potential applications in drug design and drug delivery systems.

## 3. Results

**Figure 1:**
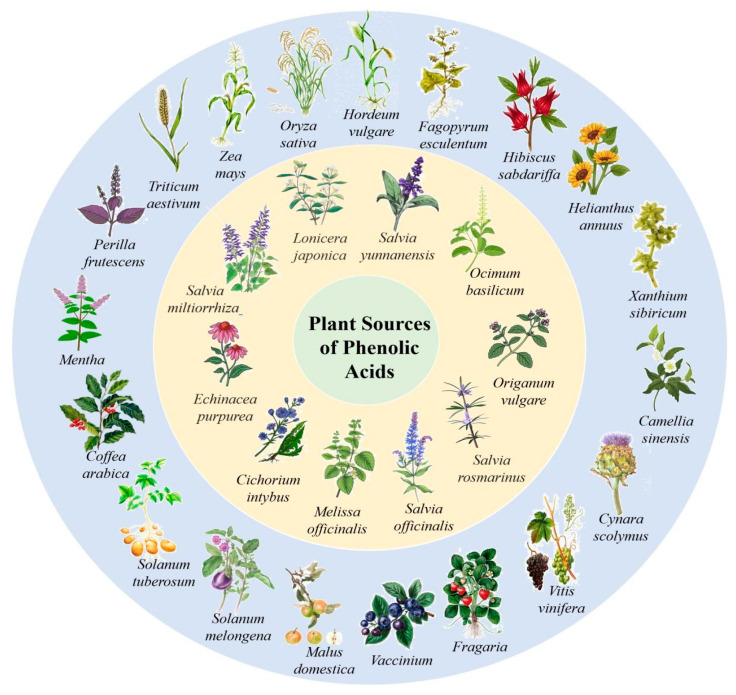
Plant sources of phenolic acids.

According to literature sources and experimental findings, typical natural sources of phenolic acids include *Lonicera japonica*, *Xanthium sibiricum*, *Salvia yunnanensis*, *Echinacea purpurea*, and other plant species (Figure 1). Table 1, Table 2 and Table 3 present the main representatives of phenolic acids and their structural classification [7]. The presented benzoic acid derivatives (Table 1) exhibit pronounced structural diversity, determined by variations in the number and position of hydroxyl, methoxy, and other substituents on the aromatic ring.

These differences influence their reactivity, acid–base properties, lipophilicity, and potential for intermolecular interactions. The comparison between two-dimensional and three-dimensional structures reveals that the position of substituents in space can affect the formation of conjugation and hydrogen bonds.

Table 1 shows that even very small structural changes can significantly alter the physical and chemical properties as well as the biological activity of benzoic acid derivatives.

The substituents on the aromatic ring and side chain of the cinnamic acid derivatives in Table 2 are different, and their conjugation degree with chlorogenic acid is also different. The structural differences of cinnamic acid derivatives will affect the electron distribution, conjugation effect and hydrogen bond formation ability, determining their chemical reactivity and biological activity. Comparison of the two-dimensional structure with the three-dimensional structure indicates that the spatial position of functional groups is very important for intermolecular interactions. The structural diversity of cinnamic acid compounds provides opportunities for chemical modification and also helps to develop more pharmacologically active derivatives.

The data analysis in Table 3 illustrates that phenolic acids are present in various plants. They can be found in medicinal plants, as well as in edible crops and feed crops.

These aromatic compounds tend to be predominantly hydroxybenzoic (C_6_–C_1_) and hydroxycinnamic (C_6_–C_3_) architectures that often exist as complex esters or polymerized complexes, notably chlorogenic and rosmarinic acids. The sources, organs and raw materials of plants vary. This indicates that these compounds have significant biological functions. They also differ in structure and function. Phenolic acids are predominantly found in leaves, roots, fruits, and seeds, with specific compounds and their concentrations depending on the plant species and the part used for study. Such systematization allows the identification of potential plant sources for further research focused on isolation, chemical modification, and investigation of the pharmacological properties of phenolic acids.

### 3.1. Chemical Characteristics of Phenolic Acids

Phenolic acids are aromatic derivatives featuring one or more hydroxyl groups alongside a carboxyl group. Based on variations in side-chain length and saturation, they fall into two main categories: hydroxybenzoic acids (C_6_–C_1_) and hydroxycinnamic acids (C_6_–C_3_), distinguished by their carbon framework, which influences their chemical behavior and biological roles. Benzoic-type phenolic acids, such as 3,4,5-trihydroxybenzoic acid; 4-hydroxy-3-methoxybenzoic acid; 3,4-dihydroxybenzoic acid; 4-hydroxybenzoic acid; and 2-hydroxybenzoic acid—are characterized by a single-carbon side chain, resulting in simpler, more stable molecular architectures. These compounds typically arise in plants from the breakdown of hydroxybenzaldehydes or aromatic alcohols. Their physicochemical traits largely stem from the positioning of hydroxyl and methoxy substituents on the benzene ring, affecting acidity, polarity, and intermolecular interactions [38].

In contrast, hydroxycinnamic-acid-derived phenolic acids possess a three-carbon side chain with a double bond directly linked to the aromatic ring, creating an extended conjugated system. Examples include (E)-3-phenylprop-2-enoic acid, (E)-3-(3,4-dihydroxyphenyl)prop-2-enoic acid, (E)-3-(4-hydroxy-3-methoxyphenyl)prop-2-enoic acid, and (E)-3-(4-hydroxyphenyl)prop-2-enoic acid. This conjugation enhances electron delocalization, contributing to high reactivity and utility in synthesizing various derivatives. Within plant systems, these compounds play key roles in polyphenol biosynthesis and serve as precursors for flavonoid and lignin production [39].

The functional attributes of phenolic acids originate from their acidic nature, redox capacity, and capacity for coordination with metal ions. The phenolic OH groups readily donate electrons, conferring potent antioxidant effects. Meanwhile, the carboxylic acid moiety allows participation in esterification, amide-bond-forming reactions, and salt-forming reactions—all crucial for enhancing solubility, metabolic stability, and oral bioavailability in pharmaceutical applications. Structure–activity relationships reveal that substitution patterns, especially hydroxyl group placement on the aromatic ring, significantly influence biological efficacy. As such, phenolic acids represent a vital class of natural products with broad relevance in chemistry and biology. Their structural diversity enables rational modification toward novel bioactive agents and advanced derivative designs [40,41].

### 3.2. Biological and Pharmacological Activity of Phenolic Acids

Phenolic acids can interact with various biological molecules. They also participate in regulating important metabolic processes, thereby possessing multiple biological activities. Many studies have found that phenolic acid derivatives of benzoic acid and cinnamic acid have antioxidant, anti-inflammatory, antibacterial, anti-tumor, neuroprotective and cardiac protective effects [42]. The various types of effects make phenolic acids have promising applications in drug research and biotechnology research.

#### 3.2.1. Antioxidant Activity

The antioxidant activity of phenolic acids is mainly related to the phenolic hydroxyl groups in their molecular structure. These functional groups can provide hydrogen atoms or electrons, thereby neutralizing reactive oxygen species (ROS) and inhibiting the occurrence of lipid peroxidation reactions [43]. The antioxidant capacity is largely influenced by the characteristics of the aromatic ring structure, especially the number and relative positions of the substituents. When there are two hydroxyl groups at the ortho or para positions, resonance effects can stabilize free radicals, while methoxy substitution helps to improve the overall stability of the molecule.

**Figure 2:**
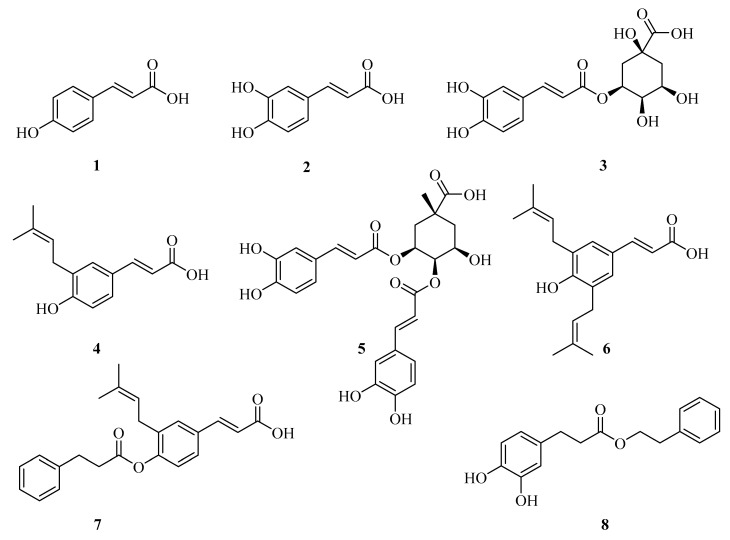
Chemical structures of the cinnamic acid derivatives in propolis components.

Among natural phenolic acids, compounds such as ferulic acid, caffeic acid, and other derivatives of cinnamic acid are well known for their antioxidant activity. Many of these molecules, including those found in propolis (Figure 2), contain phenolic hydroxyl groups that play an important role in their radical-scavenging ability [44,45]. In plants, these compounds contribute to protection against oxidative stress, while pharmacological studies have shown that they can also reduce oxidative damage in mammalian cells.

#### 3.2.2. Anti-Inflammatory and Antimicrobial Activity

The anti-inflammatory activity of phenolic acids is largely associated with their ability to modulate intracellular inflammatory pathways. A number of studies have reported that these compounds can inhibit cyclooxygenase enzymes (COX-1 and COX-2) and reduce the production of pro-inflammatory mediators such as TNF-α and IL-6 [46]. Some cinnamic acid derivatives also affect inflammatory signaling through regulation of NF-κB-related pathways. For example, caffeic acid phenethyl ester (CAPE, compound **8**; Figure 2) has been shown to suppress the phosphorylation of IκB, which leads to decreased expression of inflammation-associated proteins and contributes to its anti-inflammatory activity [45]. In addition, ferulic acid is known to inhibit prostaglandin biosynthesis, while coumaric and vanillic acids can reduce nitric oxide production in activated macrophages. Overall, these observations highlight the potential of phenolic acid scaffolds as promising candidates for the treatment of chronic inflammatory diseases [47].

**Figure 3:**
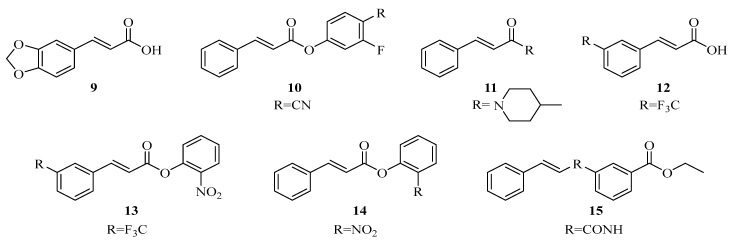
Chemical structures of cinnamic acid derivatives with antifungal activity (**9**–**15**).

In addition to their anti-inflammatory activity, phenolic acids have also been reported to exhibit antimicrobial effects. These properties are generally associated with their ability to interact with microbial cell membranes and to interfere with important metabolic enzymes. The antimicrobial activity of cinnamic acid derivatives is largely determined by the type and position of substituents on the aromatic ring. For example, several structurally modified derivatives have demonstrated antifungal activity related to inhibition of the CYP53A15 enzyme, indicating that changes in molecular structure can significantly influence biological performance (Figure 3, compounds **7**–**13**) [48].

Additional investigations suggest that electron-withdrawing groups on the aromatic framework tend to enhance activity against certain bacterial and mycobacterial strains. Moreover, naturally occurring phenolic acids such as chlorogenic acid have been described as exhibiting activity against a wide range of Gram-positive and Gram-negative microorganisms. Taken together, these observations support the view that phenolic acid derivatives possess considerable antimicrobial versatility against diverse pathogenic species [49].

#### 3.2.3. Anticancer and Neuroprotective Effects

Recent investigations suggest that phenolic acids participate in the regulation of signaling networks involved in apoptosis, cell proliferation, and angiogenesis. Caffeic acid and ferulic acid have been reported to promote programmed cell death through activation of caspases and downregulation of anti-apoptotic members of the Bcl-2 protein family. In a similar manner, gallic acid has demonstrated the ability to interfere with tumor progression by inhibiting topoisomerase activity and suppressing matrix metalloproteinases, thereby limiting metastatic potential [50,51].

The neuroprotective properties of phenolic acids are largely associated with their antioxidant capacity. By attenuating oxidative stress, maintaining mitochondrial membrane stability, and reducing β-amyloid aggregation, these compounds have attracted attention as potential agents for the prevention or management of neurodegenerative disorders such as Alzheimer’s and Parkinson’s diseases [52]. To date, various phenolic acids have shown activity against multiple cancer types, including breast, gastric, hematological, hepatic, and colorectal malignancies, among others (Table 4).

#### 3.2.4. Cardioprotective and Metabolic Effects

Phenolic acids also produce beneficial effects on the cardiovascular system. Ferulic and caffeic acids help normalize the lipid profile, lowering plasma cholesterol and triglyceride levels. Their mechanism of action is associated with enhanced activity of antioxidant enzymes and improved endothelial function of blood vessels [61].

Furthermore, these compounds also play a role in regulating glucose metabolism. They have hypoglycemic effects by inhibiting α-glucosidase and enhancing the sensitivity of tissues to insulin [62]. These properties make phenolic acids potential candidates for combined treatment of metabolic syndrome and type 2 diabetes.

### 3.3. Contemporary Approaches to the Structural Modification of Phenolic Acids

The structural optimization of phenolic acids is the cornerstone of modern drug chemistry. Its main objective is to optimize their pharmacological properties and expand their biological activity spectrum. Since these precursors are abundantly present in nature and the aromatic hydroxyl acid framework has high synthetic plasticity, phenolic acids, as flexible molecular bases, are used for the rational design and construction of novel bioactive molecules [41,63].

At present, all the modification strategies—combining physical-chemical methods, biotechnological methods and comprehensive synthetic approaches—can precisely regulate the key properties of phenolic acids, such as lipid solubility, metabolic stability, membrane permeability and receptor binding affinity. Key chemical strategies involve traditional esterification, acylation, and amidation, as well as more sophisticated interventions like hydroxymethylation or the grafting of heterocyclic and aromatic moieties. The most advanced approach currently is to design molecular hybrid structures, integrating different pharmacophores into a single framework, in order to achieve enhanced or synergistic therapeutic effects [41,64].

#### 3.3.1. Esterification and Acylation

Researchers have discovered that phenolic acids can be esterified and acylated. This process alters the polarity and lipophilicity of the molecules. After the molecular properties change, the pharmacological properties will also improve. Moreover, the molecules are less likely to be decomposed by enzymes. The resulting esters often exhibit more pronounced antioxidant and antimicrobial properties compared with the parent acids [65].

**Figure 4:**
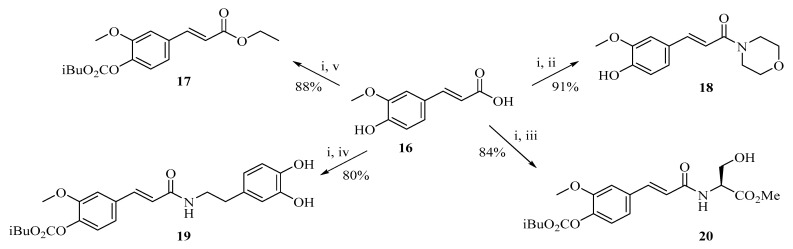
Amides and esters from ferulic acid in ethyl acetate: EtOAc, NMM, (i) iBoc-Cl, RT, 30 min, filtration; (ii) Morpholine; (iii) HCl, L-Serine methyl ester, NMM; (iv) HCl, 3,4-dihydroxy tyramine, NMM; (v) 20% DMAP in EtOH.

For example, as Figure 4 shows, the ethyl and ethyl esters of ferulic exhibited enhanced radical-scavenging activity and improved permeability across lipid membranes [66]. Introducing long-chain alkyl groups makes the molecule more soluble in lipids. This property change is crucial when preparing drugs for use on the skin.

#### 3.3.2. Amidation and Conjugation with Nitrogen-Containing Heterocycles

Amidation represents a common and effective strategy for the structural modification of phenolic acids. The formation of an amide bond can, to some extent, modulate the acidic and basic properties of the molecule while improving its chemical stability. In addition, the introduction of an amide group may alter the hydrogen-bond donor/acceptor profile and conformational behavior of the molecule, thereby influencing its biological activity. Previous studies have reported that various amide derivatives of phenolic acids exhibit antitumor, analgesic, and antibacterial activities [67].

**Figure 5:**
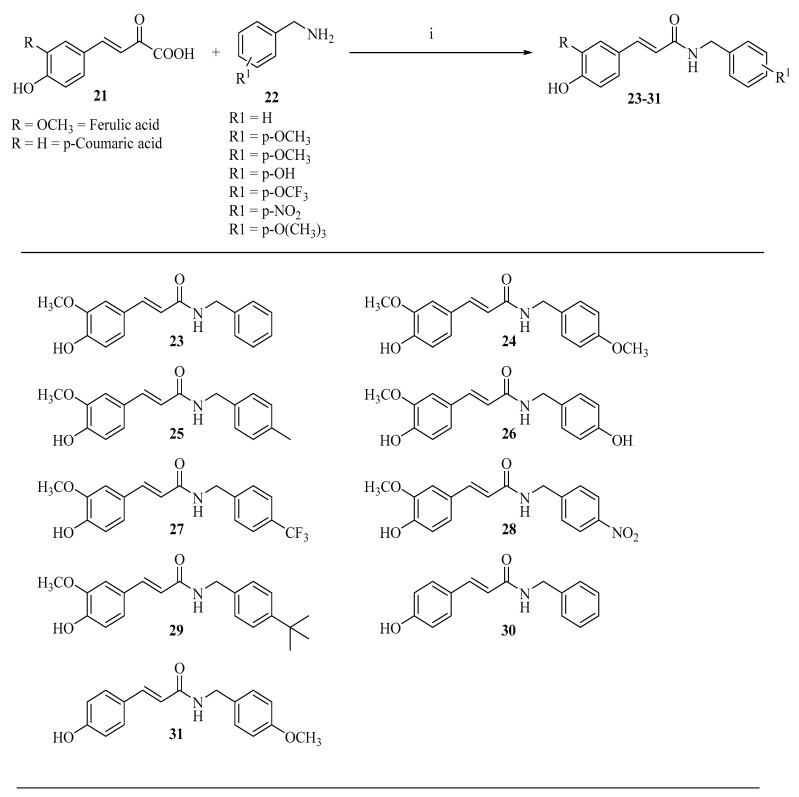
Synthesis of Cinnamoyl (Feruloyl/p-Coumaroyl) Amide derivatives (**23**–**31**): (i) DCM, ethyl chloroformate, TEA, 0 °C, 30 min; then benzylamine (1.05 equiv), TLC; workup (EtOAc, H_2_O/brine); column (EtOAc/Hexane 40:60). Yield: 40–60%.

As shown in Figure 5, cinnamoyl amide derivatives (23–31) can be obtained from ferulic acid or p-coumaric acid through an amide coupling reaction [68]. In this approach, the phenolic acid is first activated using a carbodiimide-mediated coupling system, allowing the carboxyl group to react with amine substrates and form the corresponding amide derivatives. Such structural modification may influence the physicochemical properties of the molecules, for example by improving their stability and lipophilicity, which can subsequently affect their biological activity.

**Figure 6:**
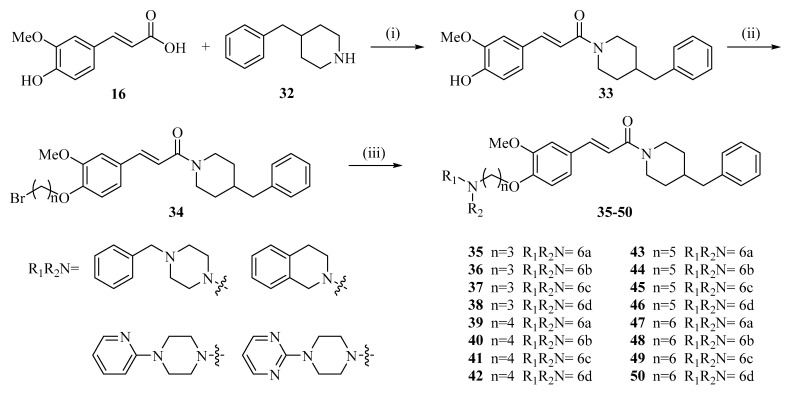
Synthesis of ferulic acid-O-alkylamines (**35**–**50**): (i) THF, EDCl, HOBT, room temperature, overnight; (ii) Br(CH_2_)nBr, CH_3_CN, reflux for 6–10 h; (iii) R1R_2_NH, K_2_CO_3_, CH_3_CN, 65 °C, for 6–10 h.

In addition, as illustrated in Figure 6, ferulic acid can be converted into ferulic acid-O-alkylamine derivatives (**35**–**50**) through nucleophilic substitution reactions with various alkylamines [69]. In some of these structures, nitrogen-containing heterocyclic fragments such as piperazine and pyridine are introduced. The incorporation of these heterocycles increases the structural diversity of the derivatives and provides additional sites that may interact with biological macromolecules.

The combination of phenolic acid scaffolds with nitrogen-containing heterocycles has led to the development of hybrid molecules that show promise for multi-target drug design. Due to their modular structural characteristics, these compounds may interact with or influence several biological pathways at the same time. Such features make them attractive candidates for further investigation in the development of therapeutic agents for complex diseases.

#### 3.3.3. Modification of the Aromatic Ring and Introduction of Substituents

**Figure 7:**
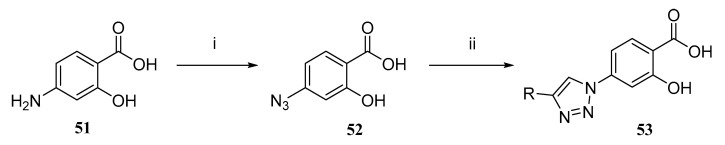
Synthesis of 4-(1,2,3-triazol-1-yl) salicylic acid derivatives: (i) NaNO_2_/H_2_SO_4_, 0 °C, 20 min; NaN_3_, H_2_O; recrystallization (EtOH), 86%; (ii) Alkyne, CuI, DIPEA, CH_2_Cl_2_, rt, 24 h; EDTA workup; recrystallization (MeOH), 43–99%.

Researchers have discovered that the properties of molecules can be altered by modifying the aromatic ring of phenolic acids. This modification can affect the electronic state and reactivity of the molecules. Replacing hydroxyl groups with methoxy, alkyl, or halogen groups can regulate the liposolubility and antioxidant capacity of the molecules. These changes will influence the interaction of the molecules with biological membranes and also affect the ability of the molecules to pass through the biological membranes [70]. For example, para-substitution usually enhances oxidative stability, while methoxy substituents increase the distribution of the molecule in the lipid phase, thereby facilitating its easier penetration of the cell barrier [71]. Moreover, as shown in Figure 7, extending the conjugated π-system within the backbone can enhance the electronic and spectral properties of the derivatives, laying the foundation for the design of more efficient therapeutic candidate molecules.

#### 3.3.4. Hybrid Molecules and Rational Design

**Figure 8:**
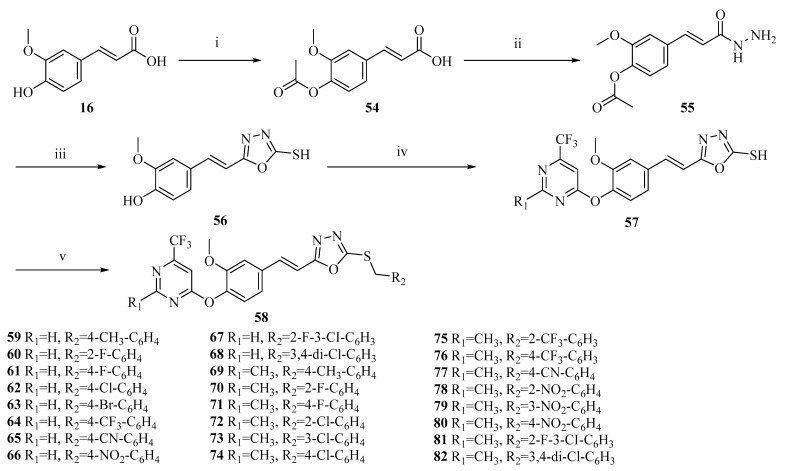
Synthetic route for ferulic acid-based hybrid derivatives (**58**–**82**): (i) (CH_3_CO)_2_O, reflux, 3 h; (ii) NH_2_NH_2_·H_2_O, EtOH, reflux, 4 h; (iii) KOH, CS_2_, MeOH/H_2_O, rt, 6 h; acidification; (iv) Cs_2_CO_3_, acetone, reflux, 8 h; (v) R_2_CH_2_Cl, NaOH, H_2_O, rt, 6 h.

One promising area in drug discovery is the design of molecular hybridization, integrating the phenolic acid backbone with other highly active biological units (such as flavonoids, alkaloids, amino alcohols, or nitrogen-containing derivatives) (Figure 8) [72]. These innovative structures are specifically designed to take advantage of pharmacological synergies, effectively integrating antioxidant, anti-inflammatory, and anti-tumor capabilities into a single therapeutic agent, thereby achieving better clinical outcomes.

By integrating and applying computer simulation strategies (such as molecular modeling and docking simulations), a precise framework has been developed to predict how the structural modifications of phenolic acids affect their binding affinity to specific enzymes and receptor targets. By accelerating the rapid screening of optimized derivatives, the time and resources consumed in the early drug discovery stages were significantly reduced, and only the most promising candidate molecules are prioritized for subsequent preclinical validation.

#### 3.3.5. Biotechnological Methods and Biocatalysis

In the contemporary field of pharmaceutical research and development, the study of structurally functionalized phenolic acids makes its transition towards biotechnological strategies more rapid. By utilizing biological catalytic systems such as lipases, esterases and peroxidases, solutions superior to traditional methods can be provided. These methods are more suitable than traditional ones. The reaction conditions are milder and energy consumption is lower. At the same time, these methods can maintain good selectivity. Microbial transformation can also synthesize some compounds that are difficult to obtain by traditional chemical methods. This approach generates fewer by-products, and the subsequent separation and purification processes are simpler [73].

These methods fully meet the fundamental principles of “green chemistry”. They provide a stable and sustainable foundation for the development of new generations of drugs.

### 3.4. Influence of Structural Modifications on the Biological Activity of Phenolic Acids

Researchers can regulate the pharmacological effects of phenolic acids by optimizing their structure. This is currently the most common and direct method. By strategically modifying the aromatic nucleus and the fatty side chain, researchers can highly directionally regulate the therapeutic spectrum of the compounds—optimizing their antioxidant, anti-inflammatory, antibacterial and anti-tumor properties [74]. The analysis of literature data and experimental studies has proved that even minor changes in the position and nature of the substituents can significantly affect the biological activity and metabolic stability of phenolic acids.

#### 3.4.1. Influence of Substituents on Antioxidant Properties

The antioxidant activity of phenolic acids is largely determined by the number and position of hydroxyl groups on the aromatic ring. Ortho- and para-dihydroxyl fragments help stabilize phenoxyl radicals through intramolecular hydrogen bonding and electron delocalization. Thus, gallic acid, which contains three hydroxyl groups, exhibits higher antioxidant activity than vanillic acid, which has only one [75].

Replacing hydroxyl groups with methoxy, alkyl, or halogen-containing fragments alters the electron density of the aromatic ring, which can enhance or diminish a compound’s ability to donate electrons [68]. Introduction of a methoxy substituent can decrease the susceptibility of the molecule to oxidation and enhance its lipophilic character, thereby facilitating membrane permeability

#### 3.4.2. Influence of the Side Chain on Bioavailability and Activity

Researchers have found that altering the structure of the phenolic side chain can affect the behavior of the drug in the body and its utilization rate [76]. When the side chain becomes longer or new double bonds are added, the molecule becomes more soluble in lipids and lighter to pass through the cell membrane. This is particularly vital for the development of oral and topical medications.

**Figure 9:**
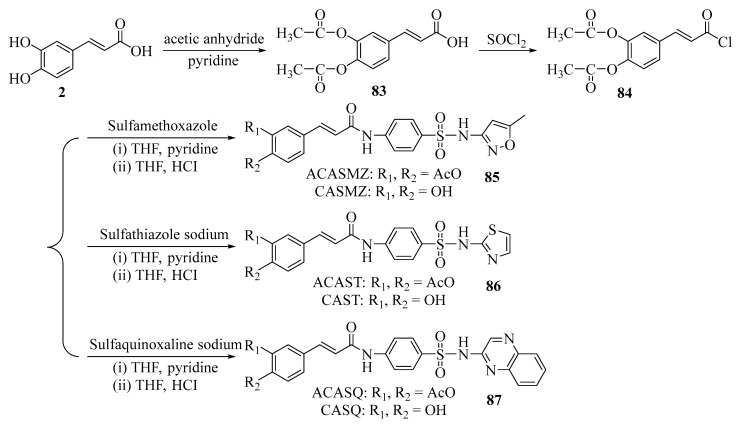
Synthesis route of caffeic acid sulfonamide derivatives.

Carboxyl groups can be esterified or amidated. This treatment alters the acid-base properties of the molecule and reduces its polarity. The molecule is less likely to be broken down by enzymes in the body. Compared to the original phenolic acid, these modified compounds have a longer duration of action and more pronounced pharmacological effects (Figure 9) [77].

#### 3.4.3. Conjugation with Heterocyclic Fragments

**Figure 10:**
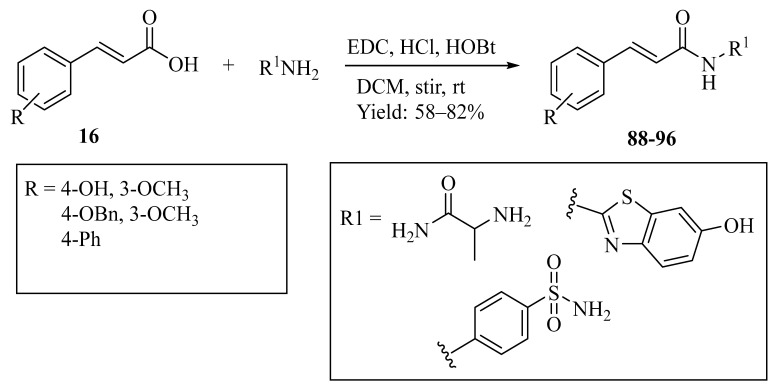
Synthesis of amide derivatives of ferulic acid.

Adding nitrogen-containing, sulfur-containing or oxygen-containing heterocyclic rings to the phenolic acid structure can significantly expand the biological activity range of the phenolic acid (Figure 10) [78].

Derivatives containing pyridine, imidazole or piperazine structures often exhibit strong antibacterial and anti-tumor effects because these compounds are more likely to form stable interactions with specific proteins [79].

In addition to their direct cellular effects, these hybrid structures also demonstrate significant potential in regulating the enzyme-driven factors that trigger oxidative and inflammatory cascades (such as cyclooxygenase and lipoxinase). This multi-mechanism action makes these derivatives ideal templates for developing new generations of antioxidant and anti-inflammatory therapies.

#### 3.4.4. Influence of Spatial Configuration and Conjugation

**Figure 11:**
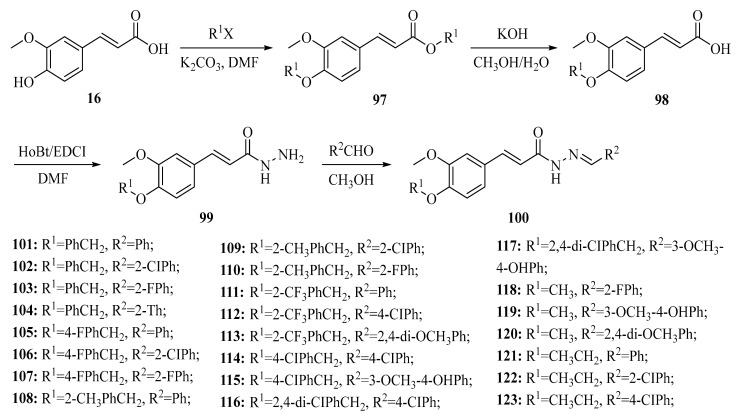
Synthetic route for ferulic acid-based hydrazone derivatives (**100**–**123**).

The biological activity of phenolic acids also depends on their three-dimensional structure and spatial configuration. As shown in Figure 11 [80], the stereochemical configuration of the side chain combined with the extended π conjugated double bond system is the main factor determining whether the molecule can effectively embed in the hydrophobic pocket of the protein receptor. These geometric features control the precise orientation of the functional groups and are the key factors for optimizing the binding affinity and ensuring the predictability of the pharmacological effects of the new derivatives. The planar structure with an extended π conjugated system can effectively interact with protein receptors and the hydrophobic regions of the membrane.

Changes in molecular geometry, for example, a transition from trans- to cis-configuration, can affect the ability to bind to enzyme active sites. Thus, the spatial orientation of functional groups is considered an important factor in designing structurally modified derivatives with predictable properties [81].

#### 3.4.5. Hybrid Structures and Synergy of Action

The creation of hybrid molecules that incorporate fragments of various biologically active compounds opens new avenues for enhancing pharmacological effects. Combining antioxidant and anti-inflammatory components within a single molecule enables pharmacodynamic synergy while reducing toxicity by lowering therapeutic doses [82].

**Figure 12:**
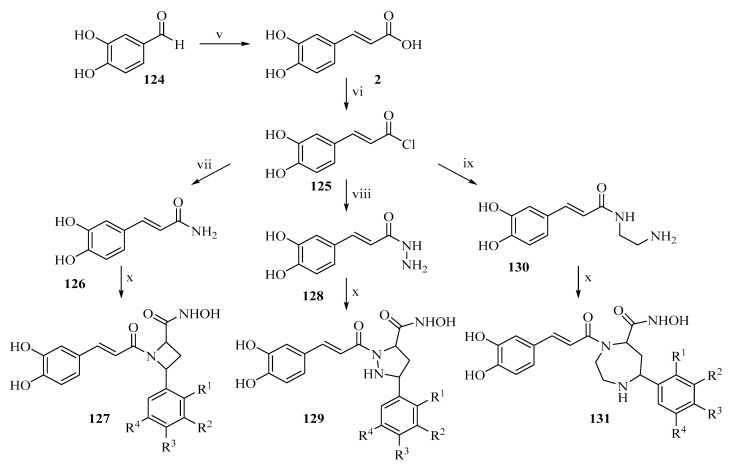
Synthetic scheme for caffeic acid derivatives: (v) Malonic acid, pvridine, 55 °C, 3 h; (vi) SOCl_2_, 4 h; (vii) NH3, 3 h; (viii) H_2_N-NH_2_, 3 h; (ix) H_2_N-(CH_2_)_2_-NH_2_, 3 h; (x) phenylbutyric acid derivatives, K_3_CO_3_, 120 °C, 90 W, Microwave, 20 min.

For example, hybrids of phenolic acids with flavonoid or alkaloid scaffolds exhibit more pronounced inhibition of tumor cell growth than the parent substances (Figure 12) [83].

Such compounds are promising prototypes for the development of multitarget drugs with combined mechanisms of action.

### 3.5. Prospects and Future Directions

Despite the significant amount of information available on the chemical and biological properties of phenolic acids, their potential as structural frameworks for the development of new therapeutic agents is still not fully explored. Most current studies are focused on structural modification of natural phenolic acids in order to overcome their known disadvantages, such as limited stability, rapid metabolic degradation, and relatively low bioavailability. Appropriate modification of the phenolic acid core often leads to noticeable changes in pharmacological activity, improved selectivity toward biological targets, and, in many cases, better therapeutic performance of the obtained derivatives.

Modern research on phenolic acid derivatives widely employs computer-aided drug design, molecular docking, and virtual screening techniques. These approaches allow prediction of possible interactions between modified molecules and biological targets before experimental testing. As a result, the early stages of drug discovery can be significantly accelerated, while the overall cost of research may be reduced. In addition, computational methods help to select the most promising structures for synthesis, which makes the search for active compounds more efficient [84,85,86,87].

In recent years, increasing attention has also been paid to biotechnological approaches for the synthesis of phenolic acid derivatives. Enzyme-catalyzed reactions involving lipases, peroxidases, and esterases make it possible to obtain new compounds under relatively mild and environmentally friendly conditions. Such methods are consistent with the principles of green chemistry and are considered especially useful in pharmaceutical research. Microbial transformation represents another valuable tool, since it can produce derivatives with unusual structures and improved biological properties that are sometimes difficult to obtain by conventional synthetic methods.

For practical medical application, it is essential to consider not only the biological activity of phenolic acid derivatives but also their pharmacokinetic properties, metabolic stability, and distribution in the organism. Many naturally occurring phenolic acids show promising activity in vitro, yet their rapid degradation and elimination in vivo often reduce their real therapeutic effect. For this reason, considerable effort is currently directed toward the development of new delivery approaches, including controlled-release systems, liposomal formulations, polymer carriers, and prodrug strategies. These technologies can enhance stability, improve bioavailability, and increase the safety of the compounds during administration.

More recently, phenolic acid derivatives have attracted attention as useful building units for modern drug delivery systems. Owing to the presence of reactive functional groups and generally good biocompatibility, phenolic acids can be incorporated into amphiphilic structures capable of forming nanosized carriers. It has been demonstrated that dendrimer-type molecules based on plant phenolic acids are able to self-assemble into stable nanoparticles suitable for the transport of biologically active substances. In particular, ionizable amphiphilic Janus dendrimers derived from phenolic acid fragments were shown to form dendrimer-like nanoparticles with high encapsulation efficiency and controllable physicochemical characteristics. Such systems are capable of delivering nucleic acids, including mRNA, to selected organs, which makes them of considerable interest for gene therapy, nanomedicine, and vaccine design. The combination of phenolic acid chemistry with modern nanocarrier technologies therefore represents a promising direction in pharmaceutical research [88,89,90,91,92,93,94,95,96,97].

Further progress in this field will likely depend on the combined use of organic synthesis, biotechnology, computational modeling, and pharmacological evaluation. Such an integrated approach makes it possible to better understand the relationship between molecular structure and biological activity and may lead to the creation of new phenolic acid derivatives with improved efficacy, higher stability, and lower toxicity. These features make phenolic acids attractive starting materials for the design of future therapeutic agents.

## 4. Conclusions

Benzoic acid derivatives and cinnamic acid derivatives are common natural compounds. They possess strong chemical reactive characteristics because their aromatic rings contain hydroxyl and carboxyl groups. This structure makes chemical modification easier. The commonly used methods in research include esterification, amidation, acylation, and the introduction of heterocyclic structures. Researchers also construct hybrid molecules containing multiple active structures. These methods can alter the properties of the molecules. They can enhance bioavailability and stability. They can also expand the drug’s action. These studies provide new directions for the development of multi-target drugs.

Phenolic acids are present in the leaves, roots, fruits and seeds of various plants. They can exist in the form of monomers, complex esters or condensates, such as chlorogenic acid and rosmarinic acid. These compounds have different structures, which affect their reactivity and the interactions between molecules. This provides a basis for studying their biological activities. Phenolic acids have various biological effects, including antioxidant, anti-inflammatory, antibacterial and anti-tumor. Through targeted chemical modifications, these effects can be enhanced, and the effects of derivatives are more concentrated. However, there are still some problems that have not been solved, such as the low utilization rate of phenolic acids in the body and the poor stability. The data on the influence of structural changes on biological activity are scattered and incomplete, highlighting the necessity of systematic knowledge integration and the development of new prodrug forms and targeted drug delivery systems.

Therefore, phenolic acids and their derivatives have significant potential in the development of innovative drugs. Comprehensive studies on their chemical properties, biological activities, and pharmacokinetic characteristics provide a solid foundation for further development in the fields of medicinal chemistry and pharmaceutical chemistry.

## Figures and Tables

**Table 1:** Chemical structure of benzoic acid derivatives and their fatty alcohol esters.

No	Compound	Chemical Structure 2D	Chemical Structure 3D
**1**	p-Hydroxybenzoic acid	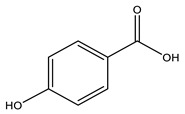	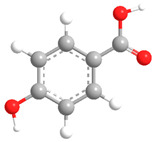
**2**	Gallic acid	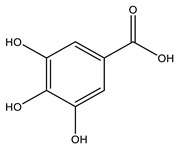	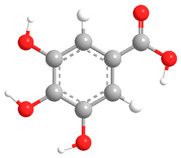
**3**	2,5-Dihydroxybenzoic acid	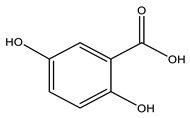	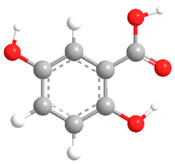
**4**	3-Hydroxy-4-methoxybenzoic acid	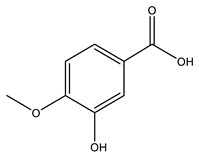	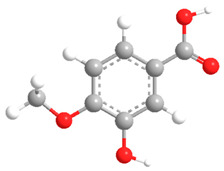
**5**	Protocatechuic acid	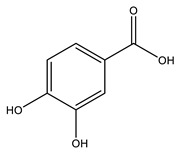	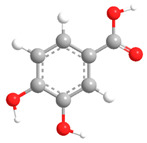
**6**	Vanillic acid	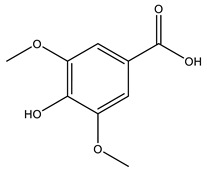	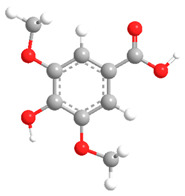
**7**	Salicylic acid	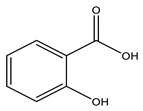	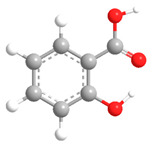
**8**	Vanillinic acid	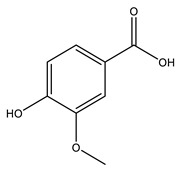	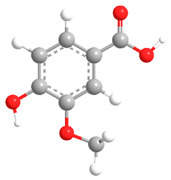
**9**	Veratric Acid	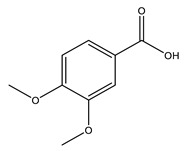	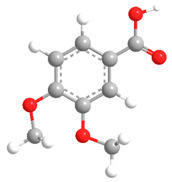

**Table 2:** Chemical structures of cinnamic acid derivatives.

No	Compound	Chemical Structure 2D	Chemical Structure 3D
**1**	p-Coumaric acid	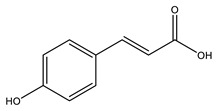	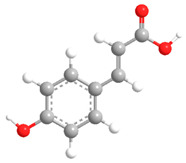
**2**	Ferulic acid	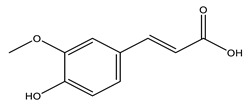	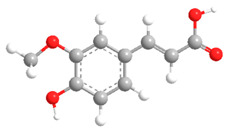
**3**	Isoferulic acid	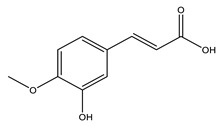	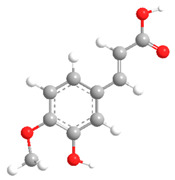
**4**	Caffeic acid	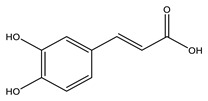	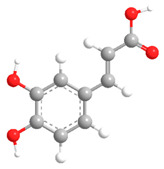
**5**	Sinapic acid	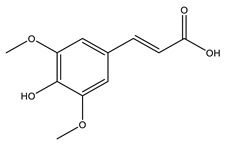	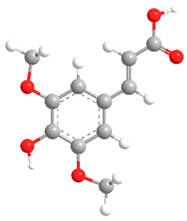
**6**	p-Coumaroylquinic acid	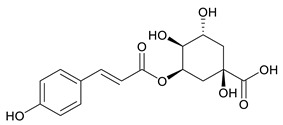	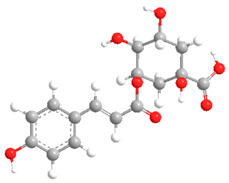
**7**	Feruloylquinic acid	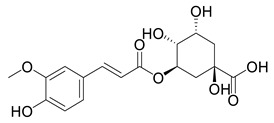	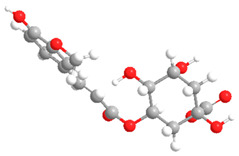
**8**	Isoferuloylquinic acid	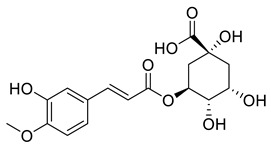	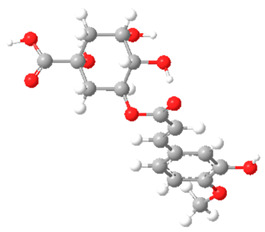
**9**	Caffeoylquinic acid	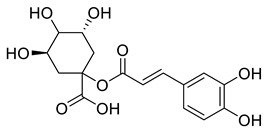	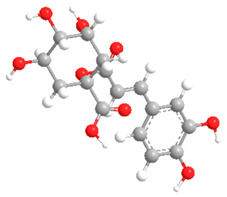
**10**	Sinapoylquinic acid	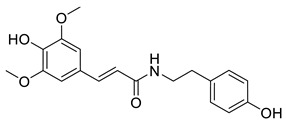	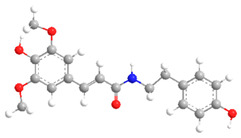

**Table 3:** Major plant sources of phenolic acids and their structural classification (C_6_–C_1_/C_6_–C_3_/derivatives)

No	Plant (Common Name)	Latin Name	Organ/Raw Material	Main Phenolic Acids (Standard Names/ English and Abbreviation)	Structural Classification (C_6_–C_1_/C_6_–C_3_/Type of Derivatives)	References
**1**	Honeysuckle	*Lonicera japonica*	Buds	Chlorogenic acids; caffeic acid	Derivatives (caffeoylquinic acids); C_6_–C_3_	[8,9]
**2**	Yunnan sage	*Salvia yunnanensis*	Roots/ rhizomes	Salvianolic acids A/B; rosmarinic acid	Derivatives (oligomers/condensed phenolic acids); C_6_–C_3_	[10]
**3**	Red sage (Danshen)	*Salvia miltiorrhiza*	Roots/ rhizomes	Salvianolic A/B; rosmarinic acid; caffeic acid	Derivatives; C_6_–C_3_	[11]
**4**	Purple coneflower	*Echinacea purpurea*	Aerial parts/roots	Chicoric acid; caffeic acid	Derivatives (dicaffeoyl tartaric esters); C_6_–C_3_	[12]
**5**	Chicory	*Cichorium intybus*	Roots/leaves	Chicoric acid; caffeoylquinic acids (CQA)	Derivatives; C_6_–C_3_	[13]
**6**	Rosemary	*Salvia rosmarinus*	Leaves	Rosmarinic acid; caffeic acid	Derivatives (diesters); C_6_–C_3_	[14]
**7**	Oregano	*Origanum vulgare*	Leaves	Rosmarinic acid; caffeic acid	Derivatives; C_6_–C_3_	[15]
**8**	Basil	*Ocimum basilicum*	Leaves	Rosmarinic acid; caffeic acid	Derivatives; C_6_–C_3_	[16]
**9**	Lemon balm	*Melissa officinalis*	Leaves	Rosmarinic acid	Derivatives	[17]
**10**	Mint	*Mentha*	Leaves	Rosmarinic acid	Derivatives	[18]
**11**	Perilla	*Perilla frutescens*	Leaves	Rosmarinic acid; caffeic acid	Derivatives; C_6_–C_3_	[19]
**12**	Common sage	*Salvia officinalis*	Leaves	Rosmarinic acid	Derivatives	[20]
**13**	Cocklebur seeds	*Xanthium sibiricum*	Fruits/ aerial parts	Caffeoylquinic acids (CQA)	Derivatives	[21]
**14**	Coffee	*Coffea arabica*	Green/roasted beans	Chlorogenic acid family (CQA family)	Derivatives	[22]
**15**	Potato	*Solanum tuberosum*	Tubers	Chlorogenic acid; caffeic acid	Derivatives; C_6_–C_3_	[23]
**16**	Eggplant	*Solanum melongena*	Fruits	Chlorogenic acid	Derivatives	[24]
**17**	Apple	*Malus domestica*	Fruits/peel	Chlorogenic acid; protocatechuic acid; p-hydroxybenzoic acid; vanillic acid	Derivatives; C_6_–C_1_	[25]
**18**	Blueberry	*Vaccinium*	Fruits	Caffeic; p-coumaric; ferulic; vanillic acids	C_6_–C_3_; C_6_–C_1_	[26]
**19**	Strawberry	*Fragaria*	Fruits	p-Coumaric; caffeic; ferulic acids	C_6_–C_3_	[27]
**20**	Grape (fruit/juice)	*Vitis vinifera*	Fruits/juice	Caftaric acid; coutaric acid	Derivatives (tartaric acid esters)	[28]
**21**	Grape (leaves)	*Vitis vinifera* (leaves)	Leaves	Caffeic; p-coumaric acids	C_6_–C_3_	[28]
**22**	Artichoke	*Cynara scolymus*	Leaves/ receptacle	CQA; dicaffeoylquinic acids (diCQA)	Derivatives	[29]
**23**	Sunflower	*Helianthus annuus*	Seed coat/ kernel	Chlorogenic acid	Derivatives	[30]
**24**	Wheat bran	*Triticum aestivum* (bran)	Bran/cell walls	Ferulic; p-coumaric acids	C_6_–C_3_	[31]
**25**	Corn bran	*Zea mays* (bran)	Bran/cell walls	Ferulic; p-coumaric acids	C_6_–C_3_	[32]
**26**	Rice bran	*Oryza sativa* (bran)	Bran/cell walls	Ferulic acid	C_6_–C_3_	[33]
**27**	Barley	*Hordeum vulgare*	Grain hull/bran	Ferulic acid	C_6_–C_3_	[34]
**28**	Buckwheat	*Fagopyrum* *esculentum*	Caryopsis/husk	Gallic; vanillic; caffeic acids	C_6_–C_1_; C_6_–C_3_	[35]
**29**	Hibiscus	*Hibiscus sabdariffa*	Calyx	Protocatechuic; caffeic acids	C_6_–C_1_; C_6_–C_3_	[36]
**30**	Tea leaves	*Camellia sinensis*	Leaves	Gallic acid (free and as galloyl residues)	C_6_–C_1_	[37]

Terminology note: CQA are caffeoylquinic acids; diCQA are dicaffeoylquinic acids; caftaric/coutaric are caffeoyl-/p-coumaroyl-tartaric acid esters.

**Table 4:** Most-studied phenolic acids with anticancer activity: cell lines and tumor types.

No	Chemical Name	Cell Lines	Cancer Types	References
1	Chlorogenic acid	MCF-7, Hep-G2	Breast cancer; hepatocellular carcinoma	[53,54]
2	Gallic acid	AGS, HL-60, MCF-7	Stomach cancer; leukemia; breast cancer	[55,56]
3	Caffeic acid	MCF-7, Hep-G2, A549, H1299, HL-60, HT-1080	Breast cancer; hepatocellular carcinoma; lung cancer; leukemia; fibrosarcoma	[54,57]
4	p-Coumaric acid	KB, HT-29, HCT-116, CAL-27, LNCaP, DU145	Colorectal cancer; oral cancer; prostate cancer	[58]
5	Ferulic acid	MCF-7, Hep-G2, HeLa, ME-180	Breast cancer; hepatocellular carcinoma; cervical cancer	[59,60]
6	3,4-Dihydroxycinnamic acid (caffeic acid)	HL-60	Leukemia	[55]
7	3,4,5-Trihydroxycinnamic acid	KB, HT-29, HCT-116, CAL-27, LNCaP, DU145	Oral cancer; colorectal cancer; prostate cancer	[58]

## Data Availability

The original contributions presented in this study are included in the article. Further inquiries can be directed to the corresponding authors.
